# MAPUNet: Multi-scale attention for InSAR phase unwrapping in mining areas

**DOI:** 10.1371/journal.pone.0331189

**Published:** 2026-05-26

**Authors:** Baojing Zhang, Wenfu Yang, Xiaowei Zhang, Li Wang, Yan Cui, Xiaoyu Wang, Yufei Zhang

**Affiliations:** 1 Shanxi Coal Geological Investigation and Research Institute CO., LTD, Taiyuan, China; 2 Key Laboratory of Monitoring and Protection of Natural Resources in Mining Cities, Ministry of Natural Resources, Jinzhong, China; Universidad de Granada, SPAIN

## Abstract

Due to the large deformation gradient caused by large-scale and high-intensity mining in the mining area, severe incoherence is prone to occur during the radar interferometry processing. The traditional phase PU method cannot obtain the correct phase unwrapping (PU) results, resulting in inaccurate monitoring results. To achieve precise monitoring of large gradient deformations in mining areas, a multi-scale phase PU method for mining area based on PUNet with multi-attention mechanism (named MAPUNet) was proposed in this paper. Firstly, the atrous spatial pyramid pooling (ASPP) is added to the original dilated block, and combined with dual attention network (DA-Net), which is used to extract the interferogram feature information at multiple scales. Then, an efficient channel attention network (ECA-Net) optimized residual module is designed to enable the network to extract important interferogram features at both branches and suppress phase noise. Three simulated interferograms with different signal-to-noise ratios (SNR) were selected for the experiment. Under conditions of large phase gradients changes and high noise (SNR = 1), compared with the optimal PUGAN method, SSIM increased by 0.0176, compared with the original PUNet model, SSIM increased by 0.298. The model in this paper has higher accuracy and better robustness, and achieves rewrapped results that are closer to the real situation in the real mining area interferogram. Finally, based on the MAPUNet model, the deformation inversion of the certain mining area in Shanxi has been achieved. The maximum settlement in this area within one month can reach −9.3 cm. Our method effectively solves the problems existing when large-scale subsidence in the mining area, providing a new technical reference for more accurate monitoring of surface deformation.

## Introduction

Coal industry has occupied the main position in China energy system for a long time. Long-term coal mining has caused widespread and increasingly severe surface subsidence, which seriously threatened the life and property safety of people and hindered the sustainable development of the mining area economy [1–4]. Therefore, it is of great scientific significance to continuously monitor the surface subsidence of mining area and analyze the patterns of subsidence variation. Surface subsidence in mining areas is generally characterized by rapid subsidence rates, large subsidence gradient, complex terrain conditions, etc. It is difficult to achieve large-scale, long-term and high-precision surface subsidence using traditional methods, such as precise leveling [5–7]. The interferometric synthetic aperture radar (InSAR) is a new type of means for monitoring surface deformation. It has the advantages of day/night data acquisition, all-weather imaging capability and strong penetrability, and has become a technical means for monitoring surface deformation in mining areas [8–10].

Phase unwrapping (PU) is a crucial step in InSAR data processing. Whether the unwrapping result is correct or not is directly related to the reliability of the final monitoring result. Therefore, many PU methods have been proposed by many scholars. In general, PU methods can be roughly divided into two categories: traditional PU methods and PU methods based on deep convolutional neural networks.

Traditional PU methods can be classified into three categories: path-following methods, optimization-based methods, and integrated denoising and PU methods [11]. Traditional PU methods are based on the assumption that the phase difference between adjacent pixels is less than π, so as to realize the process of recovering the unwrapped phase from the wrapped phase. The path-following methods use residual points or phase quality maps to set up a reasonable integral path to unwrap the wrapped phase to avoid global error transmission, such as the branched-cut method [12] and the quality graph guidance method [13, 14], etc. The basic principle of the optimization-based methods is to use different objective functions to minimize the difference between the wrapped phase gradient and the obtained estimated gradient [15,16]. Among these methods, the minimum cost flow (MCF) [17], the minimum discontinuity [18] and the least square (LS) [19,20] are the representative methods. It no longer regards phase filtering and PU as two separate steps, but adopts a simultaneous method to reduce the influence of denoising method errors on the PU process [21]. Such methods are represented by the PU method based on Kalman filter [22–24].

In recent years, with the rapid development of deep learning, some scholars have proposed some PU methods based on convolutional neural networks [25]. At present, PU based on deep convolutional neural networks (DCNN) can be roughly divided into three directions: (1) One-step PU methods, its main idea is to design a pixel-level Deep Convolutional Neural network (DCNN) and construct a direct nonlinear network prediction relationship between the wrapped phase and the unwrapped phase. Finally, a general network model for direct prediction of the interferograms was obtained [26,27]. Dardikman [28] constructed a prediction model between the unwrapped phase and the wrapped phase using the residual network (ResNet [29]), and achieved initial success. Additionally, Wang et al. [30] proposed to utilize the U-Net method to achieve unwrapping, by establishing a nonlinear mapping relationship between the wrapped phase and the true phase, further enhancing the unwrapping effect. Wu et al. [31] proposed a convolutional neural network structure (PUNet) suitable for PU, performing unwrapping on the wrapped phase. However, these methods based on U-Net are mainly designed for smooth or locally continuous phase changes, and are difficult to effectively handle the large gradients and nonlinear deformations commonly found in the center of mining subsidence basins. Moreover, they do not fully consider the coupling effect of large-scale deformations and high noise in InSAR mining monitoring, resulting in a decrease in the unwrapping accuracy in the subsidence center area and prone to unwrapping errors.

(2) Two-step PU methods, its main idea is to convert the PU of interferograms into a semantic segmentation problem, where the wrapped number of each pixel is predicted by semantic segmentation, and then processed to identify misclassified pixels [32, 33]. Wang et al. [34] conducted phase gradient estimation for interferograms to optimize the unwrapping effect. Zhang et al. [35] employed the SegNet network structure, by calculating the wrapped number of the phase and performing post-processing, to achieve unwrapping of interferograms. Compared to the one-step PU method, the two-step PU can ensure the consistency of interferometric fringes, but it is more sensitive to noise. The two-step PU method can ensure the consistency of interferometric fringes. However, for large gradients and areas where interferometric fringes are difficult to precisely identify, it is prone to significant misjudgments at the edges or in high-noise regions, thereby causing systematic deviations in subsequent phase reconstruction and limiting its robustness in large-scale subsidence areas. (3) Phase gradient estimation methods, its main idea is to combine deep learning and traditional PU methods. First, deep learning is used to estimate the phase gradient, and then the traditional PU methods are used for PU [36, 37]. This method has strict requirements for the noise and quality of the image to ensure the recognition of a clear phase gradient.

The existing PU methods have their own characteristics, and the appropriate PU methods should be selected according to the specific data characteristics and application occasions. The complex mining environment and large phase gradients will cause serious incoherence phenomenon and the interferograms will be seriously affected by noise. Therefore, interferometric fringes are prone to discontinuity and confusion. Therefore, for the case of large deformation gradients in the mining area, an appropriate PU methods should be selected, and the InSAR PU technology should be applied to the monitoring of large-scale settlement in the mining area. Compared with the traditional PU methods and the other two types of PU methods in deep learning, one-step PU methods can directly perform end-to-end PU and obtain a general network model for direct prediction of the interferograms of the mining area, which has obvious advantages.

In response to the problem of dense interferometric fringes caused by large-scale deformation in mining areas, where traditional unwrapping methods are prone to failure, this study proposes and integrates two key modules - ASPP-DA-DilatedBlock and ECA-ResidualBlock, aiming to construct a high-performance unwrapping network capable of robustly handling complex interferometric phases. ASPP-DA-DilatedBlock captures multi-scale phase features through a pyramid dilated convolution structure and combines DA-Net to enhance the focusing ability on dense fringe areas in both spatial and channel dimensions, thereby significantly improving the model’s perception of local details and global structure. ECA-ResidualBlock introduces an efficient channel attention mechanism to enhance feature selection and noise suppression capabilities, especially in low-coherence and high-noise mining area environments, effectively restoring the interferometric phases information. The combination of these two modules systematically integrates multi-scale perception and attention guidance in the phase unwrapping network, enabling the network to adaptively focus on key phase regions and suppress noise interference, thereby achieving high-precision deformation monitoring in mining areas.

Therefore, the main objectives of this paper are as follows: (1) Based on deep learning network, to explored an intelligent PU method suitable for monitoring the mine subsidence using InSAR technology; (2) To solve the problem that the PU cannot be carried out accurately in the mining area in the case of large deformaiton gradients changes; (3) In response to the settlement monitoring requirements of different types of mining areas in various regions, the PU method proposed in this paper is fully utilized to conduct PU of the interferometric fringes in the subsidence basins of mining areas, and the reliability of the model in achieving PU in different types of subsidence basins is tested.

## Material

### Data generation

In order to better improve the quality of network training and the accuracy of prediction, this paper uses simulated to construct sample datasets. The image size involved in training is 180 × 180, with a total of 2000 sets of data, which provides sample basis for deep learning training and ensures the diversity of the number and types of samples.

**Fig 1:**
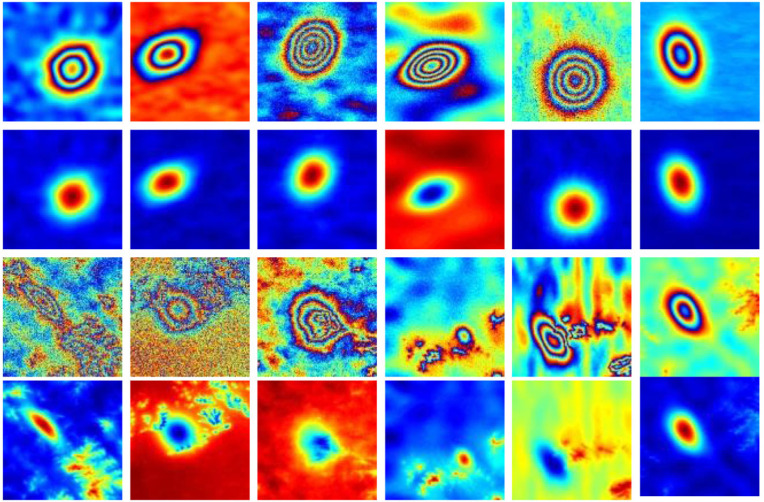
Sample dataset（partial）.


Based on the composition and principle of InSAR interferometric phase, we randomly generate 2000 pairs of the unwrapped phase labels and the noisy wrapped phases. The specific methods are as follows: The subsidence of mining area will form several elliptical subsidence basins [34]. Firstly, equation (1) is used to generate 3D subsidence surface. At the same time, the interferograms of subsidence basins with different strengths and modes can be generated by adjusting some parameters, so that the randomly generated 3D subsidence surface can be converted into the unwrapped phase. Then the unwrapped phase is wrapped according to equation (2), and different types of noises are added to obtain wrapped phase images with different SNRs. In the process of network training, the generated unwrapped phase images are used as labels for their corresponding wrapped phase images. Fig 1 is simulated InSAR mining interferogram datasets.

$$\begin{array}{c} \text{h}=\text{-}ae^{-\left(\left(\left({\left(x-x_{\text{0}}\right)}^{\text{2}}\right)/b^{\text{2}}\right)+\left(\left({\left(y-y_{\text{0}}\right)}^{\text{2}}\right)/c^{\text{2}}\right)\right)} \end{array}$$ (1)

$$\begin{array}{c} wrap=mod\left(\frac{val\times 4\pi }{\lambda }+\pi ,2\pi \right)-\pi \end{array}$$ (2)

In equation (1), h is the settlement; (x, y) is the plane coordinates of settlement points; (x_0_, y_0_) is the position of maximum subsidence center in the plane coordinate system; a is the influence of the subsidence factor; b and c are the semimajor axis and semiminor axis of an elliptic equation, respectively. In equation (2), *mod* is the modulo operation; *val* is the elevation value of the 3D surface Z; λ is the radar wavelength; *wrap* is the wrapped phase.

To simulate the real interferogram environment with low coherence and high noise in the monitoring of mining area deformation, we systematically introduced two types of noise when generating synthetic training dataset: one is additive complex Gaussian noise controlled by random SNR (SNR ∈ [−1.01 dB, −1 dB]), which is used to simulate thermal noise and de-coherence effects; the other is spatially correlated atmospheric phase perturbation generated based on fractal Perlin noise, with its amplitude controlled within the range of [−π, π] radians. Both types of noise are applied to the interferogram generation process in complex form – the variance of the main noise is dynamically adjusted according to the sample SNR, while the atmospheric noise is directly superimposed onto the real phase. This strategy effectively reproduces challenging scenarios such as vegetation coverage, time de-coherence, and atmospheric delay, providing practical training and testing conditions for the PU algorithm.

### Experimental data

**Fig 2:**
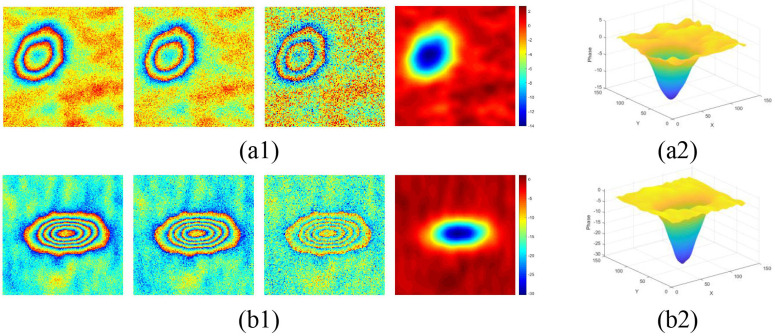
Simulated interferograms. (a1) is the wrapped phases with small phase gradients with different noise; (b1) is the wrapped phases with large phase gradients with different noise. From left to right, some noise is added with SNR = 8, 4, 1 in each Fig. respectively; (a2) and (b2) are the corresponding three-dimensional display maps.

Two kinds of simulated interferograms with different phase gradients are selected to carry out PU experiments under different SNRs, and the size of the test image is 180 × 180. Fig. 2(a1) is the wrapped phases with small phase gradients and the corresponding unwrapped phases are within the range of 0–20 rad. Under three different SNRs, Fig. 2(a2) is the three-dimensional display maps of the unwrapped phase. Fig. 2(b1) is the wrapped phases with large phase gradients under three different SNRs and the corresponding unwrapped phases are within the range of 30–50 rad, and Fig. 2(b2) is the three-dimensional display maps of the unwrapped phase (Fig 2).

### Real mining area data

#### Location of the study area.

**Fig 3:**
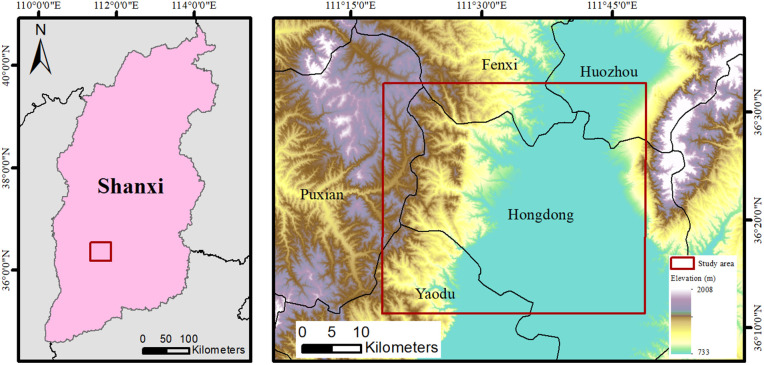
The location of the research area in a certain mining area in Shanxi Province.

This mining area is mainly located near Hongtong County, Linfen City, Shanxi Province. The region is dominated by mountains and hills and has a warm temperate continental climate. As shown in Fig 3, the red box is the main study area of this paper. The subsidence basin is mainly located at the junction of Hongtong County and Huozhou City, Puxian County, Fenxi County, and Yaodu District.

#### Image data.

2 Sentinel-1A images of a certain mining area in Shanxi Province from November 2020 to December 2020 were selected to form interferometric pair for PU experimental test. The information of interferometric pairs is shown in Table 1.

**Table 1:** Information of Sentinel-1A interferometric pairs.

No.	Master image	Slave image	Relative orbit	Frame	Temporal baseline(d)	Spatial baseline(m)
**1**	12/16/2020	01/09/2021	113	116	24	63.1

Sentinel-1 is an Earth observation satellite in the Copernicus program of the European Space Agency. It consists of two satellites (1A and 1B), which were launched in 2014 and 2016 respectively. The scanning width of the Interferometric Wide format mode (IW) of Sentinel-1 is 250 km. Currently, Sentinel-1 satellite SAR data is freely accessible to global users.

#### Preprocessing.

The experiment utilized D-InSAR technology, employing a 30-meter resolution Shuttle Radar Topography Mission (SRTM) Digital Elevation Model (DEM) to eliminate the flat terrain effect, and applying Goldstein phase filtering to the differential interferogram to suppress decorrelation noise. Subsequently, individual subsidence basins within the study area were located and cropped from the original SAR imagery, generating corresponding small-area interferograms for each. Finally, the proposed MAPUNet method was applied to unwrap the interferometric phases of each subsidence basin, yielding high-precision continuous phase results.

## Methods

During large-scale subsidence in mining areas, the discontinuous or partially confused interferometric fringes in mining area are easy to occur under the situation of low coherence or high SNR. In order to make the network model pay more attention to the PU of complex region, based on the basis of PUNet model, a multi-scale phase PU method for mining area based on PUNet with multi-attention mechanism (MAPUNet) was constructed in this paper. The ASPP module is introduced to extract multi-scale interferograms information. The multi-attention mechanism makes it pay more attention to significant feature information in interferograms and suppresses the influence of noise. To further explore the advantages of this PU method in different scenarios, a certain mining area in Shanxi Province was selected as the typical experimental area respectively. According to the difference in the clarity of interferometric fringes in the subsidence basin in the mining area, the reliability of MAPUNet PU and deformation inversion was tested.

### ASPP-DA-DilatedBlock

**Fig 4:**
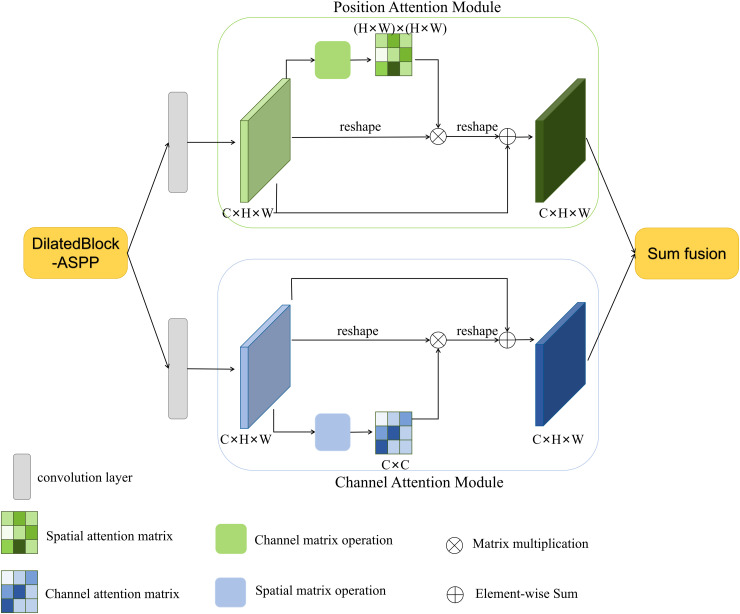
ASPP-DA-DilatedBlock model structure diagram.

Since large-scale subsidence in mining area will result in dense interferometric fringes, the unwrapping errors are easy to occur. To solve this problem, the dilated block that combines ASPP [38] and DA-Net [39] is used to capture multi-scale information of fringe-dense regions in interferograms and improve the PU performance of the network. Firstly, a multi-scale dilated convolution module was designed. Drawing on the scheme of the pyramid convolution module in the Deeplab V3 semantic segmentation network, the expansion rate of the dilated convolution was designed as a pyramid type to capture the feature information under different receptive fields and obtain the semantic information at different scales. The module structure is shown in Fig 4.

At the same time, in order to make the network pay more attention to the feature information of the fringe-dense region in the interferograms, the DA-Net is proposed to integrate the output of the ASPP feature maps. DA-Net combines spatial attention and channel attention, and its purpose is to enhance the module’s attention to specific spatial positions and channels. So that it can better capture important information about the local context of the fringe-dense regions in interferograms. The spatial attention mechanism in the DA-Net module uses a 3 × 3 convolutional kernel in the spatial domain, generating a sigmoid activation mapping ranging between 0 and 1. This mapping is applied to each pixel position in the input feature map to emphasize important spatial regions. The channel attention mechanism in this module employs global average pooling, convolution, and the Sigmoid function along the channel dimension to generate the corresponding activation weight mapping for channel-wise feature calibration. This mapping is then used to weight each channel in the input feature map, highlighting crucial channel-specific information. The spatial and channel attention mechanisms in the DA-Net contribute to a better understanding of the relationships between pixels in the image, particularly when dealing with SAR interferograms containing complex information. The design of the DA-Net allows the network to incorporate spatial attention when dealing with local structures, while simultaneously enhancing awareness of global context through channel attention. In the phase unwrapping of SAR interferograms, this design enables the network to better handle local details and global phase structures, and help to improve the accuracy of unwrapping.

### ECA-ResidualBlock

ECA-Net is an efficient channel attention module designed for deep CNNs. It is essentially an improvement upon the SE-Net module, introducing a non-reductive local cross-channel interaction strategy (ECA module) and an adaptive selection method for one-dimensional convolution kernel size. This approach leads to performance enhancements without dimension reduction, providing an effective means of optimizing performance. ECA-Net module can effectively make the network pay more attention to the features that are helpful for the current task, improving the perception ability of network and suppressing noise in the feature maps. Compared with the ordinary residual block, ECA-ResidualBlock module can learn image features more effectively, suppress the negative impacts of irrelevant noise, and improve the accuracy of the network.

**Fig 5:**
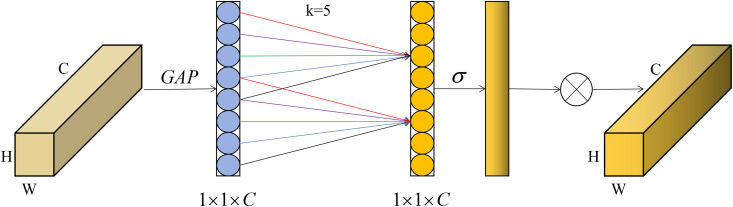
ECA-ResidualBlock model structure diagram.

In the case of low coherence or high noise level, the problem of phase confusion or partial discontinuity is easy to occur in the interferograms of mining area. Therefore, in order to better suppress noise, improve the ability of the network to extract useful features, and optimize the final unwrapping results, the ECA-Net [40] is proposed to add the original residual block to recover the feature information of the interferograms. The module structure is shown in Fig 5. In this module, a convolutional layer is first passed through, and then the output of the convolutional layer is normalized to improve the stability and convergence speed of the network. Then the output of the convolutional layer is nonlinear activated by relu activation function. The ECA-Net is added to the convolutional layer, batch normalization (BN) and relu processing of residual block to weight channel features, improve important channel weights in the feature map, and suppress irrelevant noise features.

### MAPUNet

**Fig 6:**
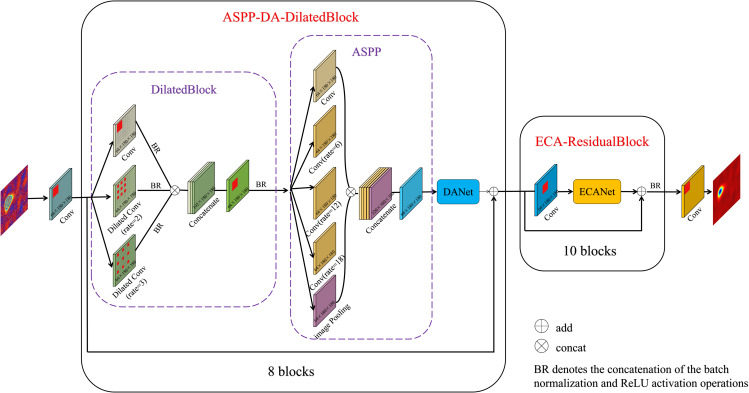
An illustration of the MAPUNet model architecture.

As shown in Fig 6, the MAPUNet network proposed in this paper is used to achieve the direct mapping from the interferogram to the unwrapped phase. Table 2 presents the structure of the MAPUNET model. In the first layer of MAPUNet, three convolution kernels of different sizes with dilation rates of 1,2, and 3 are first used to form pyramid dilated convolution, which is used to capture multi-scale information and balance the number of parameters, especially for capturing spatial features under different dilation rates. The ASPP module is designed with different-sized convolutional kernels with dilation rates of 1, 6, 12, and 18, aiming to capture features at different spatial receptive fields. This design further enhances the ability of network to perceive multi-scale information from the input interferograms. After the processing of the two modules, the double attention mechanism involving both spatial and channel attention (DA-Net) is applied, enabling the network to enhance sensitivity to the phase information in the interferograms. At the second layer of MAPUNet, the residual block fused with ECA-Net is mainly used to recover the feature information. Each residual block consists of a single convolutional layer, followed by channel weighting through ECA-Net, ultimately completing the weighting of feature information.

**Table 2:** The architecture table of the MAPUNet model.

Module	Type	Input Channels	Output Channels	Kernel Size
**Input**	layer	1	1	/
**Conv1**	conv	1	64	3*3
**Conv2−1**	conv	64	64	3*3
**Dilated Conv2−2(rate = 2)**	conv	64	64	3*3
**Dilated Conv2–3(rate = 3)**	conv	64	64	3*3
**Concatenate1**	layer	192	192	/
**Conv2**	conv	192	64	3*3
**ASPP Conv3−1**	conv	64	64	3*3
**ASPP Conv3−2(rate = 6)**	conv	64	64	3*3
**ASPP Conv3−3(rate = 12)**	conv	64	64	3*3
**ASPP Conv3–4(rate = 18)**	conv	64	64	3*3
**Conv3–5(image Pooling)**	conv	64	64	1
**Concatenate2**	layer	320	320	/
**Conv3**	conv	64	64	1
**DANet**		64	64	/
**Conv4**	conv	64	64	3*3
**ECANet**		64	64	/
**Conv5**	conv	64	2	3*3
**Output**	layer	2	2	/

### Supplement details

#### Accuracy Evaluation Index.

The difference mean (M) and mean square error (MSE) are selected to evaluate the performance of the proposed method and other PU methods. M and MSE can be expressed by equations (3) and (4) respectively.


$$\begin{array}{c} M=\frac{\text{1}}{mn}\sum_{i=\text{1}}^{m}\sum_{j=\text{1}}^{n}|I(i,j)-K(i,j)| \end{array}$$ (3)

$$\begin{array}{c} MSE=\frac{\text{1}}{mn}\sum_{i=\text{1}}^{m}\sum_{j=\text{1}}^{n}|I(i,j)-K(i,j){|}^{\text{2}} \end{array}$$ (4)

where, *I(i, j)* is the unwrapping result of the method; *K(i, j)* is the real phase; m and n are the number of rows and columns of the image respectively.

At the same time, we select the structural similarity index (SSIM) [41] to comprehensively evaluate the quality of unwrapping results. The SSIM can be expressed as equation (5). SSIM is an index used to measure the similarity of two images. The larger the SSIM, the smaller the difference between the two images, and the better the PU performance of method. The maximum value of SSIM is 1. SSIM can be used to measure the structural similarity between two images.


$$\begin{array}{c} SSIM\left(x,y\right)={\left[l\left(x,y\right)\right]}^{\alpha }{\left[c\left(x,y\right)\right]}^{\beta }{\left[s\left(x,y\right)\right]}^{\gamma } \end{array}$$ (5)

$$\begin{array}{c} \begin{array}{c} l\left(x,y\right)=\frac{2\mu_{x}\mu_{y}+C_{\text{1}}}{\overset{\text{2}}{\underset{x}{\mu }}+\overset{\text{2}}{\underset{y}{\mu }}+C_{\text{1}}}\\ c\left(x,y\right)=\frac{2\sigma_{x}\sigma_{y}+C_{\text{2}}}{\overset{\text{2}}{\underset{x}{\sigma }}+\overset{\text{2}}{\underset{y}{\sigma }}+C\text{2}}\\ s\left(x,y\right)=\frac{\sigma_{xy}+C_{\text{3}}}{\sigma_{x}\sigma_{y}+C_{\text{3}}} \end{array} \end{array}$$

Where, *x* and *y* are reference images and images to be evaluated respectively; l(x,y), c(x,y) and s(x,y)are the luminance, contrast and structure comparison measures of *x* and *y*; $\mu_{x}$ and $\mu_{y}$ are the mean of *x* and the mean of *y*; $\sigma_{x}$ and $\sigma_{y}$ are the variance of *x* and *y* respectively; $\sigma_{xy}$ is the covariance of *x* and *y*; *C*_*1*_, *C*_*2*_ and *C*_*3*_ are small constants; α, β and γ are weight ratio.

#### General experiment settings.

The experimental training platform employed Python 3.9 and Pytorch 1.1.3 deep learning framework, with GPU and CUDA11.7 for training and acceleration. The total number of image pairs with size 180 × 180 is 2000, which consists of 1600 training image pairs and 400 test image pairs. During the training process, batch_size was set to 2, Epoch to 300, and MSE and Adam were respectively used as the loss function and gradient optimization method. Additional settings of the training platform are shown in Table 3. In addition, based on the original platform settings, additional detailed information about the core training configuration, optimizer configuration, and learning rate scheduler of the current model (MAPUNet) and the baseline model (ResUNet) has been added. Table 4 presents the model parameters and inference time for various deep learning-based PU models.

**Table 3:** Network training platform.

	Parameter category	MAPUNet	Baseline model（ResUNet）
**Network platform settings**	Processor	i7-9750H CPU @ 2.60GHz	i7-9750H CPU @ 2.60GHz
	RAM	16.0 G	16.0 G
	GPU	NVIDIA GeForce RTX 2070	NVIDIA GeForce RTX 2070
	Video memory	8.0 GB	8.0 GB
	Operating system	Windows 10 64 bit	Windows 10 64 bit
**Core training configuration**	Total number of training epochs	300	300
	Batch size	2	2
	Loss function	MSELoss	L1Loss
**Optimizer configuration**	Optimizer	AdamW	Adam
	Initial learning rate	1e × 10−4	1e × 10−4
	Weight decay	1e × 10−3	1e × 10−3
	Momentum parameter	0.9	0.9
**Learning rate scheduler**	Scheduler type	CosineAnnealingWarmRestarts	CosineAnnealingWarmRestarts
	T-0	20	20
	T-mult	2	2
	Eta-min	1e × 10−6	1e × 10−6

**Table 4:** Model parameters and inference time.

Model	Model parameters	Inference time
**ResUNet**	26114881	0.0870
**UNet++**	36628676	0.0606
**PUGAN**	28878594	0.0469
**PUNet**	2142017	0.0212
**MAPUNet**	3602096	0.0338

## Results and discussion

In this section, two types of experiments are presented to evaluate the performance of the newly constructed PU method. On the one hand, by using the simulated interferograms, it is proved that the proposed method has better PU performance for interferograms with different phase gradients under different SNRs. On the other hand, the unwrapping results of different types of original interferograms in mining areas are evaluated and analyzed. In both cases, we compared the performance of the new method with those of Quality Guide PU (QGPU), Least Squares (LS), Minimum Cost Flow (MCF), ResUNet [42],UNet++ [43], PUGAN [25] and SegNet PU methods [35].

The noise of different SNRs will affect the final results of the PU methods in different degree, but noise is not the only concern in the PU process. Different phase gradients will also affect the PU process and the final unwrapping results. Therefore, in order to evaluate the effectiveness of the new method proposed in this paper, we first divided the simulated interferograms into two groups according to the density of the wrapped phase: (1) The interferogram with small phase gradients, whose range is about 0–20 rad; (2) The interferogram with large phase gradients, whose range is about 30–50 rad. Then, referring to relevant literature [44], we divided three different SNRs: SNR = 8 was classified as low noise, SNR = 4 was classified as medium noise, and SNR = 1 was classified as high noise. Next, experiments were conducted on simulated data with different phase gradients under different SNRs. The unwrapping results were compared and analyzed with those of three representative traditional PU methods and ResUNet, UNet++, PUGAN, SegNet PU and original PUNet method to demonstrate the PU performance of the proposed method.

### Evaluation and Analysis of PU in Simulated Interferograms

#### Interferogram with small phase gradients.

**Fig 7:**
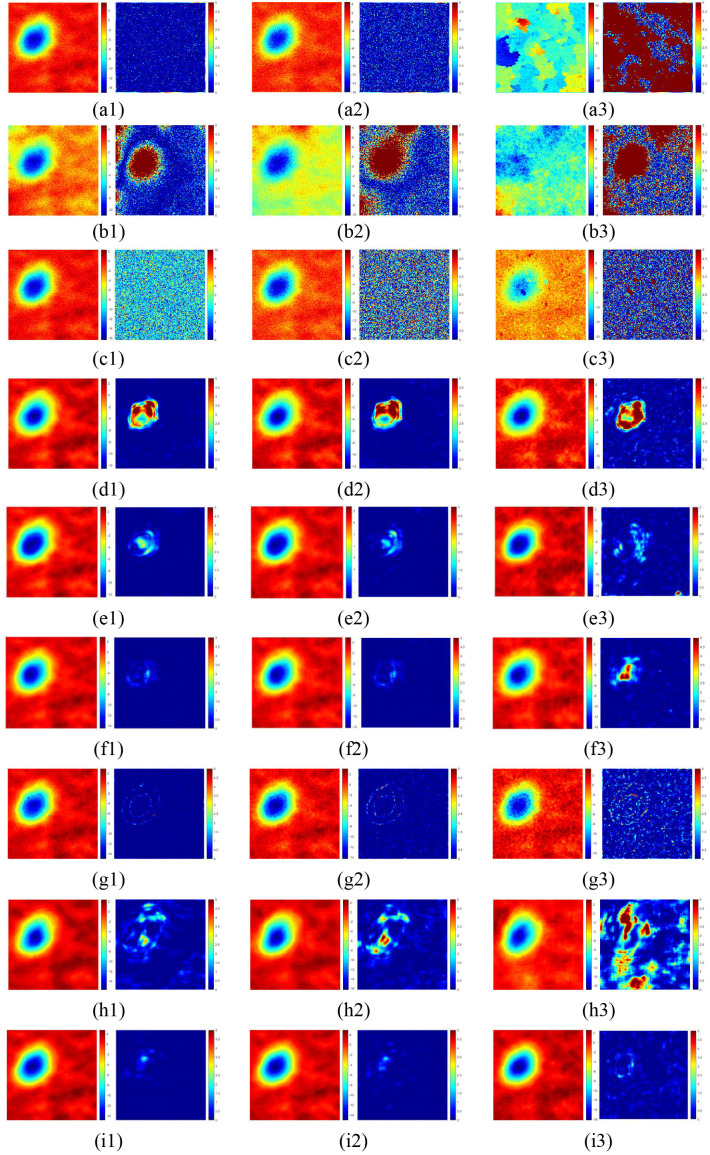
The unwrapping results of interferogram with small phase gradients: (a1) – (a3), (b1) – (b3), (c1) – (c3), (d1) – (d3), (e1) – (e3), (f1) – (f3), (g1) – (g3), (h1) – (h3), (i1) – (i3) are the unwrapping results and the error distribution maps of QGPU, LS, MCF, ResUNet, UNet++, PUGAN, SegNet PU, PUNet and MAPUNet methods under three different noise levels (SNR = 8, SNR = 4, and SNR = 1), respectively.

For Interferogram with small phase gradients, the wrapped phase image and the unwrapped phase image under low, medium and high noise levels are shown in Fig. 2 (a1). Under three different noise levels, the unwrapping results of the nine methods are shown as (a1 - a3) – (i1 - i3) in Fig 7. The unwrapping errors of the LS, ResUNet, and UNet++ methods are primarily concentrated in regions with large deformations. The errors of QGPU and MCF are evenly distributed throughout the entire image. The unwrapping error distribution of the original PUNet is wider compared with the improved MAPUNet. The error of the SegNet PU method is concentrated at the edge of the subsidence basin. Therefore, among these nine unwrapping methods, the PUGAN model and the MAPUNet model perform relatively better.

The nine PU methods are quantitatively evaluated, as shown in Table 5 (5-fold cross validation results of deep learning methods are shown in S1 Appendix). The best results are marked in bold. As the noise level increases, the performance of the traditional PU method significantly deteriorates, while the PU method based on deep learning demonstrates a notable advantage in noise resistance. Under three different SNR conditions, the PUGAN and MAPUNet models outperform the other unwrapping models based deep learning overall. In low- and medium-noise scenarios, PUGAN achieves slightly better unwrapping metrics than MAPUNet; however, under high-noise conditions, the proposed MAPUNet model demonstrates superior unwrapping performance compared to PUGAN. Compared with the original PUNet, the M and MSE of the MAPUNet are reduced by 0.6474 and 1.004 respectively, and the SSIM is increased by 0.454. Quantitative evaluation shows that, when unwrapping simulated interferograms with small deformations under high-noise conditions, the MAPUNet model preserves the original structural features as much as possible during the unwrapping process. Its overall performance surpasses that of the other eight PU methods.

**Table 5:** The quantitative evaluation indexes of nine PU methods for interferogram with small phase gradients under three different noise levels (SNR = 8, SNR = 4, and SNR = 1).

	Method	M	MSE	SSIM
**SNR = 8**	QGPU	0.4887	0.3473	0.4862
	LS	0.9614	1.7026	0.2995
	MCF	1.3136	1.9367	0.1268
	ResUNet	0.3416	0.3506	0.8138
	UNet++	0.1915	0.1092	0.8873
	PUGAN	0.1402	0.0446	0.9008
	SegNet PU	0.1711	0.0603	0.7741
	PUNet	0.3846	0.2473	0.6892
	MAPUNet	0.1538	0.0476	0.8915
**SNR = 4**	QGPU	0.5413	0.4627	0.3739
	LS	1.3931	3.3248	0.1593
	MCF	1.2716	2.0133	0.1416
	ResUNet	0.3626	0.3864	0.7579
	UNet++	0.1893	0.0905	0.8563
	PUGAN	0.1545	0.0491	0.8651
	SegNet PU	0.2373	0.1024	0.672
	PUNet	0.4493	0.3389	0.6404
	MAPUNet	0.1666	0.0533	0.8634
**SNR = 1**	QGPU	6.9573	87.2789	0.0002
	LS	2.1956	9.3153	0.0235
	MCF	1.1761	2.5153	0.1557
	ResUNet	0.4863	0.5302	0.543
	UNet++	0.3274	0.2004	0.6486
	PUGAN	0.2962	0.1984	0.7158
	SegNet PU	0.4645	0.3598	0.4115
	PUNet	0.9355	1.2016	0.2982
	MAPUNet	0.2881	0.1976	0.7522

#### Interferogram with large phase gradients.

**Fig 8:**
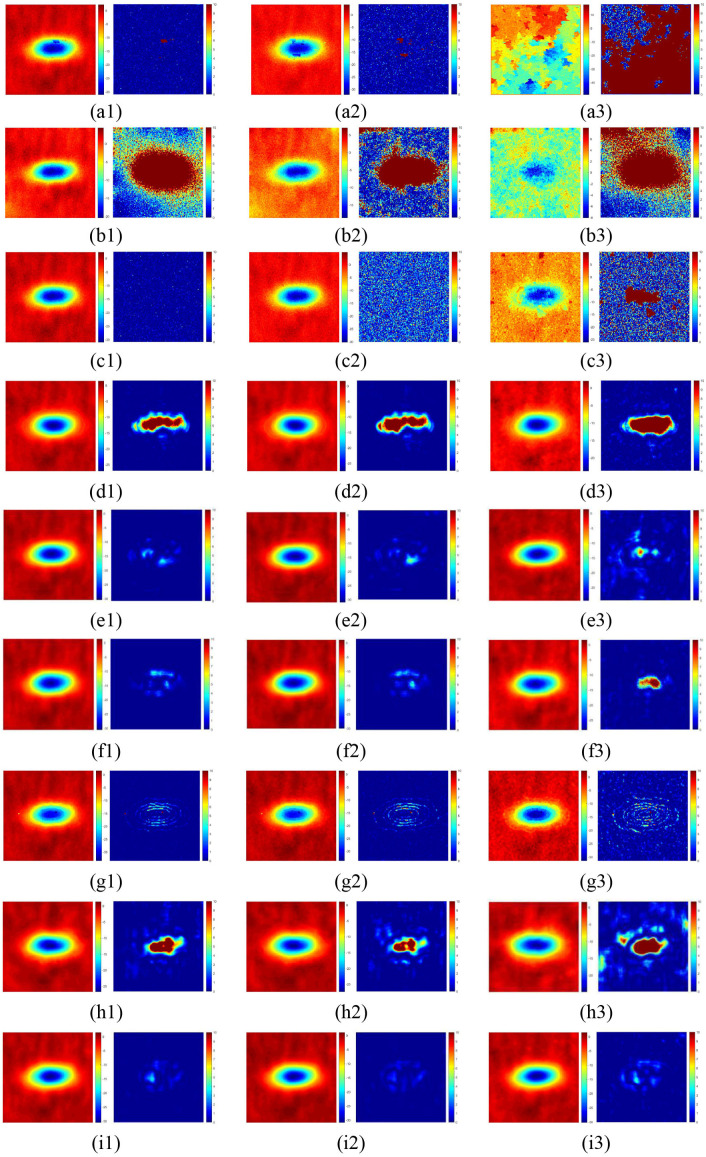
The unwrapping results of interferogram with large phase gradients: (a1) – (a3), (b1) – (b3), (c1) – (c3), (d1) – (d3), (e1) – (e3), (f1) – (f3), (g1) – (g3), (h1) – (h3), (i1) – (i3) are the unwrapping results and the error distribution maps of QGPU, LS, MCF, ResUNet, UNet++, PUGAN, SegNet PU, PUNet and MAPUNet methods under three different noise levels (SNR = 8, SNR = 4, and SNR = 1), respectively.

For Interferogram with large phase gradients, the wrapped phase image and the unwrapped phase image under low, medium and high noise levels are shown in Fig. 2 (b1). Under three different noise levels, the unwrapping results of the seven methods are shown as (a1 - a3) – (i1 - i3) in Fig 8. The distribution area of the unwrapping error of LS is the largest. The errors of QGPU and MCF are evenly distributed over the entire image. The SegNet PU method exhibits more pronounced errors at the edges of the subsidence basin. The unwrapping errors of ResUNet, UNet++, and PUGAN are primarily concentrated in the regions of maximum deformation. Compared with the other eight unwrapping methods, the proposed MAPUNet method demonstrates a relatively smaller error distribution area overall, achieves better overall phase recovery, and shows superior unwrapping performance.

For interferogram with large phase gradients, the nine PU methods are quantitatively evaluated (5-fold cross validation results of deep learning methods are shown in S1 Appendix). In Table 6, the quantitative evaluation indexes of the unwrapping results of the interferogram under the three different SNRs shown in Fig 8 are respectively recorded. Because the phase gradients are large, the unwrapping errors of all methods exhibit corresponding increases under equivalent noise levels. When the phase change is large, the errors of the traditional methods are significant. The ResUNet and original PUNet methods perform well compared to traditional methods, but the errors are obvious in areas with dense interferometric fringes. The overall error level of SegNet PU is relatively small under the three noise levels, but it is affected by the noise at the fringes. The PUGAN method performed relatively well overall. However, the MAPUNet method achieved the lowest M and MSE values, the highest SSIM value, the smallest error, and the smoothest result. The MAPUNet method has the smallest and smoothest visual error, the lowest indicators on M and MSE, and a higher indicator on SSIM. In the case of high noise, the M and MSE of the unwrapping results of the MAPUNet method are 0.2866 and 0.166 respectively. The overall unwrapping error is relatively small. Under different SNRs, the SSIM values for the proposed method consistently exceed 0.75, outperforming any other model. Therefore, for the interferogram with large phase gradients, the proposed method can easily extract more useful features to protect the structure, and obtain satisfactory results, which is generally superior to other methods.

**Table 6:** The quantitative evaluation indexes of nine PU methods for interferogram with large phase gradients under three different noise levels (SNR = 8, SNR = 4, and SNR = 1).

	Method	M	MSE	SSIM
**SNR = 8**	QGPU	0.3939	0.3300	0.5388
	LS	2.8136	11.8893	0.0138
	MCF	0.3849	0.2326	0.5193
	ResUNet	0.4679	0.8341	0.8143
	UNet++	0.1997	0.1068	0.8767
	PUGAN	0.2148	0.1051	0.8837
	SegNet PU	0.2209	0.133	0.8045
	PUNet	0.7872	1.0233	0.6032
	MAPUNet	0.2112	0.0958	0.8877
**SNR = 4**	QGPU	0.5698	0.7502	0.4206
	LS	3.1621	15.3680	0.0176
	MCF	1.2447	1.9433	0.1928
	ResUNet	0.4849	0.96	0.775
	UNet++	0.239	0.1325	0.8173
	PUGAN	0.2255	0.1156	0.8623
	SegNet PU	0.2806	0.1803	0.7042
	PUNet	0.8533	1.1197	0.5604
	MAPUNet	0.2172	0.0933	0.8637
**SNR = 1**	QGPU	10.2320	172.4367	0.0272
	LS	3.2160	31.5820	0.0141
	MCF	1.7038	6.0571	0.1534
	ResUNet	0.71	2.0062	0.6084
	UNet++	0.4446	0.3529	0.588
	PUGAN	0.3364	0.334	0.7451
	SegNet PU	0.4963	0.4296	0.4696
	PUNet	1.1295	1.7903	0.4646
	MAPUNet	0.2866	0.166	0.7627

### Application examples of MAPUNet in mining areas

#### PU of the subsidence basins in a certain mining area of Shanxi Province.

The experimental data were selected from multiple subsidence basins on two Sentinel-1A images of a certain mining area in Shanxi Province during the winter of 2020–2021. The Sentinel-1A images in summer were not selected for the experiment. This decision was made because the vigorous vegetation growth during this period hampers the ability of SAR images to perform effective interferometric processing. In contrast, the interferograms of the mining area in winter is clearer and easier to interpret than that in other seasons. However, due to the influence of human activities, geological factors and surface changes, etc., in winter, the mining area may still experience a relatively serious degree of confusion in interferograms.

**Fig 9:**
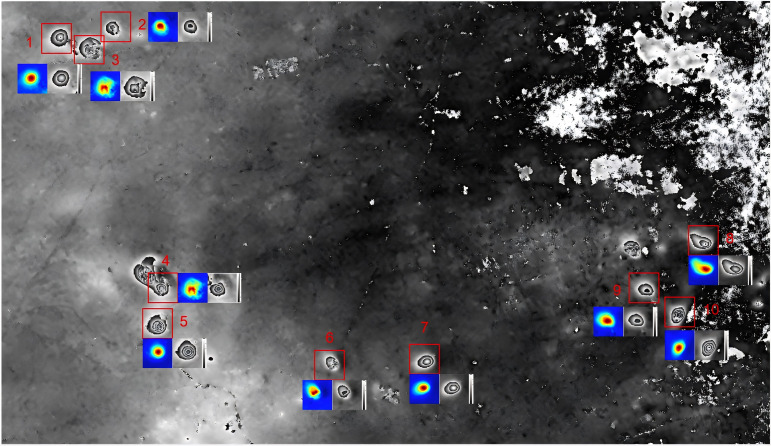
Phase interferogram.

As shown in Fig 9, the final phase distribution map of the mining area is obtained. The interferometric phase results of the mining area from December 16, 2020 to January 9, 2021 are presented in Fig 9. The red boxes indicate the locations of multiple subsidence basins, and the numbers of each subsidence basin are also marked respectively in the figure. The newly constructed MAPUNet in this paper was adopted to unwrap multiple subsidence basins in this area. The map shows the unwrapping results of multiple subsidence basins and their rewrapped results. Finally, the subsidence values of the mining areas during this period were obtained based on the unwrapping results.

**Fig 10:**
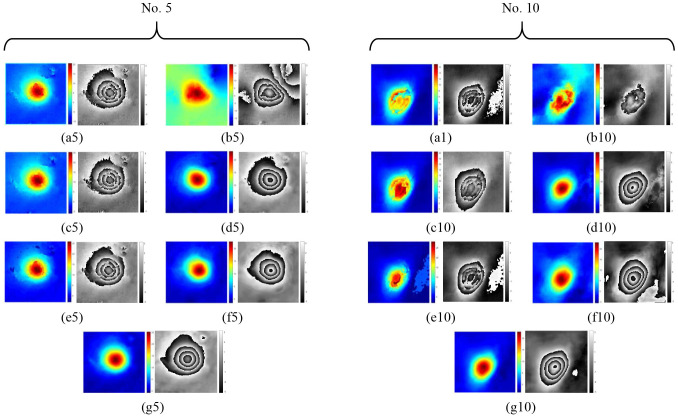
The PU results and rewrapped results of mining areas No. 5 and No. 10. ((a5,a10), (b5,b10), (c5,c10), (d5,d10), (e5,e10), (f5,f10), (g5,g10) are the corresponding results of QGPU, LS, MCF, ResUNet, SegNet PU, PUNet and MAPUNet, respectively).

Two representative subsidence basins (No. 5 and No. 10) were selected from multiple subsidence basins for detailed analysis. Phase unwrapping was performed using seven different methods: QGPU, LS, MCF, ResUNet, SegNet PU, original PUNet and the MAPUNet. The unwrapping results were subsequently rewrapped. As shown in Fig 10, the MAPUNet yields the most accurate unwrapping results. Moreover, its rewrapped phase appears smoother and more consistent with the original interferometric phase compared to the other methods.

#### PU of the subsidence basins in Hami mining area of Xinjiang Province.

**Fig 11:**
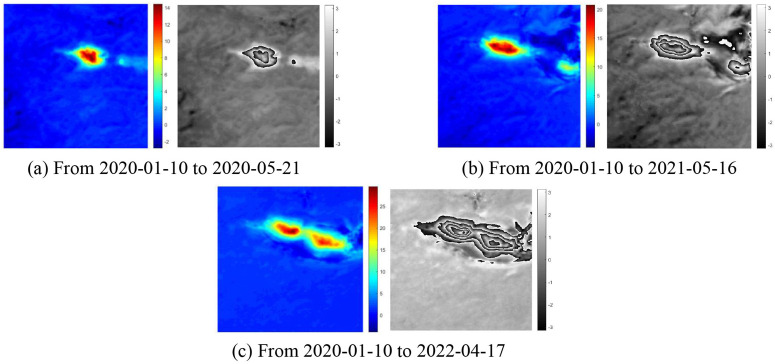
The unwrapping and rewrapped results of Hami mining.

To improve the model’s generalization and practicality, we also used interferograms from the Hami mining area for PU experiments. Fig 11 shows the unwrapped and rewrapped phase results for the mining area across three time periods. The specific data information of the Hami mining area can be found in S2 Appendix. Here, only the unwrapping results are presented. As can be seen from (a) to (c), the proposed method successfully unwraps the phase for the Hami mining area. The unwrapped results were rewrapped, and the interferograms of the subsidence basins in the resulting wrapped image were clear and continuous. This proves that the method proposed in this paper can effectively solve the problem of severe confusion of interferometric fringes in subsidence basins of the long temporal baselines in the Hami mining area, and achieves high-quality unwrapping results.

#### Inversion of settlement.

**Fig 12:**
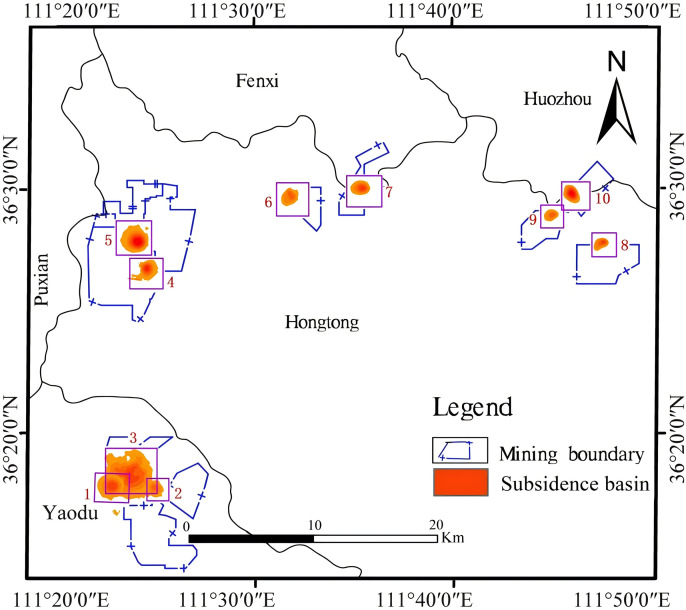
Thematic map of subsidence.

Based on the unwrapping results, subsequent processing steps including phase-to-deformation conversion and geocoding were performed. The subsidence thematic maps of the mining area during this period were respectively drawn, displaying only those subsidence basins where the subsidence exceeded −1 cm. It can be seen from the thematic map that the distribution of subsidence locations is relatively scattered. In addition, when overlaid with the mine boundary vector data of Linfen City, it can be clearly observed that all subsidence areas identified in this study fall entirely within the official mining boundary. As shown in Fig 12, the settlement range of mining area No. 3 is relatively large, and the maximum settlement in this area is 5.89 cm. It can be concluded from Table 7 that the settlement of the No. 5 and No. 10 subsidence basins are the largest, with the maximum settlement of the two subsidence basins being 9.30 cm and 8.39 cm respectively.

**Table 7:** The maximum settlement of each subsidence basin.

Subsidence basin	Maximum settlement/cm	Subsidence basin	Maximum settlement/cm
**Number 1**	−5.64	Number 6	−4.26
**Number 2**	−4.00	Number 7	−5.79
**Number 3**	−5.89	Number 8	−5.44
**Number 4**	−7.00	Number 9	−4.10
**Number 5**	−9.30	Number 10	−8.39

### Ablation Experiment

**Fig 13:**
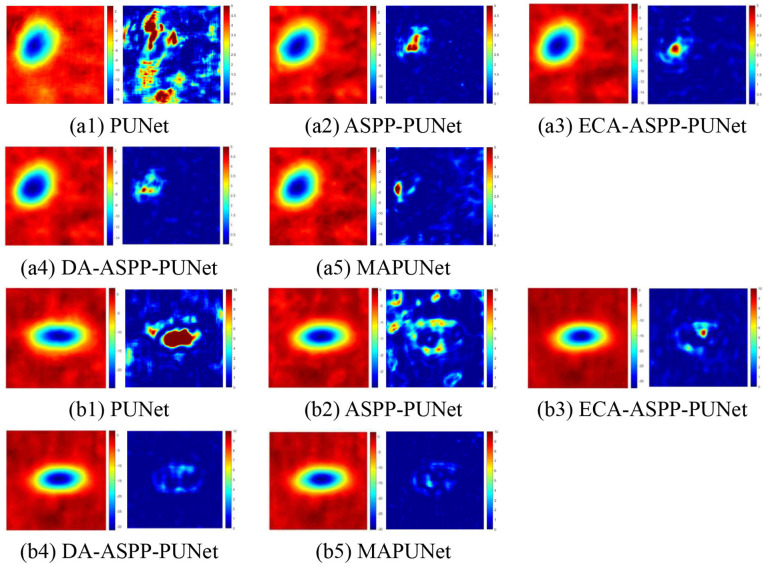
Phase unwrapping results and error distribution of five ablation models (PUNet, ASPP-PUNet, ECA-ASPP-PUNet, DA-ASPP-PUNet and MAPUNet) under high noise.

To verify the effectiveness of each ablation module, five ablation models (PUNet, ASPP-PUNet, ECA-ASPP-PUNet, DA-ASPP-PUNet and MAPUNet) were respectively constructed. Fig 13 shows the PU results of different phase gradients of the wrapped phase at a high noise level (SNR = 1 db). The wrapped phase with smaller phase changes and the real images are shown as (a1) – (a5) in Fig 13; the wrapped phase with larger phase changes and the real images are shown as (b1) – (b5) in Fig 13; the quantitative evaluation of the PU results under different SNR is shown in Tables 8 and 9.

**Table 8:** The quantitative evaluation indexes of five ablation models small with large phase gradient.

	Method	M	MSE	SSIM
**SNR = 8**	PUNet	0.3846	0.2478	0.6889
	ASPP-PUNet	0.2890	0.1749	0.7879
	ECA-ASPP-PUNet	0.1927	0.0778	0.8779
	DA-ASPP-PUNet	0.1609	0.0489	0.8803
	MAPUNet	**0.1538**	**0.0475**	**0.9035**
**SNR = 4**	PUNet	0.4493	0.3389	0.6405
	ASPP-PUNet	0.3381	0.2604	0.7449
	ECA-ASPP-PUNet	0.2086	0.0874	0.8584
	DA-ASPP-PUNet	0.1734	0.0588	0.8672
	MAPUNet	**0.1674**	**0.0454**	**0.8826**
**SNR = 1**	PUNet	0.9354	1.2009	0.2985
	ASPP-PUNet	0.3724	0.2259	0.7440
	ECA-ASPP-PUNet	0.3012	0.1856	0.7711
	DA-ASPP-PUNet	0.2797	0.1742	0.7623
	MAPUNet	**0.2760**	**0.1333**	**0.7883**

**Table 9:** The quantitative evaluation indexes of five ablation models with large phase gradient.

	Method	M	MSE	SSIM
**SNR = 8**	PUNet	0.7868	1.0224	0.6025
	ASPP-PUNet	0.4945	0.7665	0.7130
	ECA-ASPP-PUNet	0.3828	0.2314	0.7844
	DA-ASPP-PUNet	0.2497	0.1680	0.8913
	MAPUNet	**0.2113**	**0.0959**	**0.8977**
**SNR = 4**	PUNet	0.8537	1.1215	0.5605
	ASPP-PUNet	0.4746	0.6036	0.6881
	ECA-ASPP-PUNet	0.4403	0.3299	0.7548
	DA-ASPP-PUNet	0.2733	0.1924	0.8658
	MAPUNet	**0.1785**	**0.0598**	**0.8636**
**SNR = 1**	PUNet	1.1298	1.7912	0.4646
	ASPP-PUNet	0.8317	1.6181	0.4785
	ECA-ASPP-PUNet	0.4854	0.3802	0.6882
	DA-ASPP-PUNet	0.3250	0.2134	0.7669
	MAPUNet	**0.2866**	**0.1659**	**0.7826**

As can be seen from Fig 13, under high noise levels, regardless of whether the range of phase gradients changes is large or small, with the improvement of the model and the addition of each module, the accuracy of the model’s PU has been improved. As clearly shown in Fig.13 (a1)–(a5) and (b1)–(b5), under relatively high noise levels, the MSE gradually decreases as the model is progressively improved. Among all ablation models, MAPUNet method produces the most continuous and smooth unwrapping results, and its error distribution covers a smaller region compared to the other four ablation models. Based on both the unwrapping results and error distribution maps, we conclude that MAPUNet not only effectively performs PU but also efficiently suppresses phase noise in interferometric fringes, thereby achieving the optimal unwrapping performance.

Tables 8 and 9 show that, under varying noise levels and for interferograms with different phase variation ranges, the MAPUNet model consistently achieves the lowest values in terms of M and MSE, and the highest value in SSIM. Its unwrapping results also exhibit the smallest deviation from the corresponding true phases, demonstrating that MAPUNet outperforms the previously proposed models in unwrapping performance. Notably, even under high noise conditions and for large deformaiton gradients changes of mining areas, MAPUNet maintains robust performance—producing phase results that closely approximate the true phase while effectively preserving original structural features without significant deviations. Furthermore, comparisons among ablation variants confirm that each newly introduced module plays an indispensable role in enabling the final MAPUNet model to achieve its superior unwrapping results.

## Conclusion

To address the issue of inaccurate monitoring results in mining areas caused by large deformation gradients and noise interference during PU, this paper constructs a multi-scale phase PU method for mining area based on PUNet with multi-attention mechanism(MAPUNet) and applies it to the settlement monitoring of mining areas in different scenarios. Experiments were conducted by selecting simulated interferograms with different deformation gradients and real mining area scenarios. Compared with other traditional and deep learning PU methods, MAPUNet is less affected by noise levels and deformation gradients. Under conditions of large deformation gradient changes and high noise, it achieves the best performance in the three indicators of M, MSE and SSIM.

For the simulated interferograms with different phase gradients, compared with other methods, the PU accuracy of the proposed method is less affected by noise or phase gradients. This indicates that the PU performance of the proposed method is superior than that of the original PUNet model in subsidence basin with large phase gradients under the condition of large phase gradients changes and high noise. Compared with the optimal MCF in traditional methods, M and MSE decreased by 1.4172 and 5.8911 respectively, and SSIM increased by 0.6093. Compared with the PUGAN, the M and MSE decreased by 0.0498 and 0.168, respectively, and the SSIM increased by 0.0176. The proposed method has better performance on accuracy and stronger noise resistance.

Through the rewrapped analysis in a real case of a certain mining area in Shanxi Province and a mining area in Hami, the MAPUNet results were smoother and closer to the original interferometric phase. The monthly maximum settlement of −9.3 cm in this mining area was successfully monitored using MAPUNet. Our method effectively addresses the problems existing when large-scale settlement PU in mining areas, providing a new technical reference for more accurate monitoring of surface deformation and demonstrating good practicability. However, the absence of leveling data for auxiliary validation remains a limitation. In the future, we will combine the on-site measurement results to further evaluate the reliability and advantages of MAPUNet in more scenarios.

## Supporting information

S1 Appendix Location of the Hami study area. (DOCX)

S2 Appendix The unwrapping and rewrapped results of Hami mining. (DOCX)

S3 Appendix The deep learning five-fold cross-validation results of small phase gradients. (DOCX)

S4 Appendix The deep learning five-fold cross-validation results of large phase gradients. (DOCX)

S5 Appendix The deep learning five-fold cross-validation results of small phase gradients. (DOCX)

S6 Appendix The deep learning five-fold cross-validation results of large phase gradients. (DOCX)

S7 Appendix Information of Sentinel-1A interferometric pairs. (DOCX)
