# Supplementary material for: MAPUNet: Multi-scale attention for InSAR phase unwrapping in mining areas
Source: PLoS One. 2026 May 26;21(5):e0331189. doi: 10.1371/journal.pone.0331189 (PMC13210142; doi:10.1371/journal.pone.0331189)
Supplement: S1 Appendix — (DOCX) [file pone.0331189.s001.docx]

# **S1 Appendix-The deep learning five-fold cross-validation results of small phase gradients.**

**S1 Table 1. The deep learning five-fold cross-validation results of small phase gradients.**

| Noise | Method | K-fold | M | MSE | SSIM |
| --- | --- | --- | --- | --- | --- |
| SNR=8 | ResUNet | 1^st^ fold | 0.3398 | 0.3485 | 0.8121 |
|  |  | 2^nd^ fold | 0.3432 | 0.351 | 0.815 |
|  |  | 3^rd^ fold | 0.3405 | 0.3492 | 0.8143 |
|  |  | 4^th^ fold | 0.3441 | 0.3531 | 0.8117 |
|  |  | 5^th^ fold | 0.3406 | 0.3512 | 0.8159 |
|  |  | Mean±std | 0.3416±0.0019 | 0.3506±0.0018 | 0.8138±0.0018 |
|  | UNet++ | 1^st^ fold | 0.1902 | 0.1085 | 0.8856 |
|  |  | 2^nd^ fold | 0.1931 | 0.1102 | 0.8881 |
|  |  | 3^rd^ fold | 0.192 | 0.1094 | 0.887 |
|  |  | 4^th^ fold | 0.1917 | 0.1088 | 0.8892 |
|  |  | 5^th^ fold | 0.1905 | 0.1091 | 0.8865 |
|  |  | Mean | 0.1915±0.0012 | 0.1092±0.0007 | 0.8873±0.0014 |
|  | PUGAN | 1^st^ fold | 0.1391 | 0.0439 | 0.9021 |
|  |  | 2^nd^ fold | 0.141 | 0.0452 | 0.8996 |
|  |  | 3^rd^ fold | 0.1404 | 0.0447 | 0.9012 |
|  |  | 4^th^ fold | 0.1418 | 0.0455 | 0.8989 |
|  |  | 5^th^ fold | 0.1387 | 0.0438 | 0.9023 |
|  |  | Mean | 0.1402±0.0013 | 0.0446±0.0008 | 0.9008±0.0015 |
|  | SegNet PU | 1^st^ fold | 0.1703 | 0.0598 | 0.7724 |
|  |  | 2^nd^ fold | 0.1721 | 0.0609 | 0.7758 |
|  |  | 3^rd^ fold | 0.1714 | 0.0602 | 0.7735 |
|  |  | 4^th^ fold | 0.1699 | 0.0597 | 0.7749 |
|  |  | 5^th^ fold | 0.172 | 0.0611 | 0.774 |
|  |  | Mean | 0.1711±0.001 | 0.0603±0.0006 | 0.7741±0.0013 |
|  | PUNet | 1^st^ fold | 0.3825 | 0.2451 | 0.6903 |
|  |  | 2^nd^ fold | 0.3861 | 0.249 | 0.6878 |
|  |  | 3^rd^ fold | 0.3842 | 0.2472 | 0.6895 |
|  |  | 4^th^ fold | 0.3857 | 0.2484 | 0.6882 |
|  |  | 5^th^ fold | 0.3845 | 0.2469 | 0.6901 |
|  |  | Mean | 0.3846±0.0014 | 0.2473±0.0015 | 0.6892±0.0011 |
|  | MAPUNet | 1^st^ fold | 0.1525 | 0.0469 | 0.8926 |
|  |  | 2^nd^ fold | 0.1543 | 0.0481 | 0.8907 |
|  |  | 3^rd^ fold | 0.1536 | 0.0474 | 0.8919 |
|  |  | 4^th^ fold | 0.1551 | 0.0485 | 0.8898 |
|  |  | 5^th^ fold | 0.1535 | 0.047 | 0.8923 |
|  |  | Mean | 0.1538±0.001 | 0.0476±0.0007 | 0.8915±0.0012 |
| SNR=4 | ResUNet | 1^st^ fold | 0.3612 | 0.3847 | 0.7561 |
|  |  | 2^nd^ fold | 0.3638 | 0.3875 | 0.759 |
|  |  | 3^rd^ fold | 0.3621 | 0.3852 | 0.7583 |
|  |  | 4^th^ fold | 0.364 | 0.3889 | 0.7572 |
|  |  | 5^th^ fold | 0.3619 | 0.3857 | 0.7588 |
|  |  | Mean | 0.3626±0.0012 | 0.3864±0.0018 | 0.7579±0.0012 |
|  | UNet++ | 1^st^ fold | 0.1881 | 0.0899 | 0.8552 |
|  |  | 2^nd^ fold | 0.1905 | 0.0912 | 0.8571 |
|  |  | 3^rd^ fold | 0.1894 | 0.0906 | 0.8565 |
|  |  | 4^th^ fold | 0.1899 | 0.091 | 0.8558 |
|  |  | 5^th^ fold | 0.1887 | 0.09 | 0.8569 |
|  |  | Mean | 0.1893±0.0009 | 0.0905±0.0006 | 0.8563±0.0008 |
|  | PUGAN | 1^st^ fold | 0.1532 | 0.0485 | 0.8664 |
|  |  | 2^nd^ fold | 0.1551 | 0.0496 | 0.8642 |
|  |  | 3^rd^ fold | 0.1546 | 0.0492 | 0.8653 |
|  |  | 4^th^ fold | 0.156 | 0.05 | 0.8638 |
|  |  | 5^th^ fold | 0.1535 | 0.0483 | 0.866 |
|  |  | Mean | 0.1545±0.0012 | 0.0491±0.0007 | 0.8651±0.0011 |
|  | SegNet PU | 1^st^ fold | 0.2355 | 0.1013 | 0.6708 |
|  |  | 2^nd^ fold | 0.2382 | 0.103 | 0.6732 |
|  |  | 3^rd^ fold | 0.237 | 0.1021 | 0.672 |
|  |  | 4^th^ fold | 0.2391 | 0.1038 | 0.6715 |
|  |  | 5^th^ fold | 0.2368 | 0.1019 | 0.6727 |
|  |  | Mean | 0.2373±0.0014 | 0.1024±0.001 | 0.672±0.001 |
|  | PUNet | 1^st^ fold | 0.4472 | 0.3365 | 0.642 |
|  |  | 2^nd^ fold | 0.4501 | 0.3398 | 0.6395 |
|  |  | 3^rd^ fold | 0.4488 | 0.3382 | 0.6412 |
|  |  | 4^th^ fold | 0.451 | 0.3409 | 0.6389 |
|  |  | 5^th^ fold | 0.4494 | 0.3391 | 0.6403 |
|  |  | Mean | 0.4493±0.0014 | 0.3389±0.0017 | 0.6404±0.0013 |
|  | MAPUNet | 1^st^ fold | 0.1651 | 0.0524 | 0.8647 |
|  |  | 2^nd^ fold | 0.1672 | 0.0538 | 0.8625 |
|  |  | 3^rd^ fold | 0.1664 | 0.053 | 0.8638 |
|  |  | 4^th^ fold | 0.1681 | 0.0542 | 0.8619 |
|  |  | 5^th^ fold | 0.1662 | 0.0529 | 0.8642 |
|  |  | Mean | 0.1666±0.0011 | 0.0533±0.0007 | 0.8634±0.0012 |
| SNR=1 | ResUNet | 1^st^ fold | 0.4845 | 0.5281 | 0.545 |
|  |  | 2^nd^ fold | 0.4872 | 0.5314 | 0.5412 |
|  |  | 3^rd^ fold | 0.4861 | 0.5297 | 0.5436 |
|  |  | 4^th^ fold | 0.488 | 0.533 | 0.5408 |
|  |  | 5^th^ fold | 0.4858 | 0.5289 | 0.5445 |
|  |  | Mean | 0.4863±0.0013 | 0.5302±0.002 | 0.543±0.0019 |
|  | UNet++ | 1^st^ fold | 0.3258 | 0.1991 | 0.6502 |
|  |  | 2^nd^ fold | 0.3284 | 0.201 | 0.6475 |
|  |  | 3^rd^ fold | 0.327 | 0.2002 | 0.6489 |
|  |  | 4^th^ fold | 0.3291 | 0.2021 | 0.6468 |
|  |  | 5^th^ fold | 0.3267 | 0.1995 | 0.6496 |
|  |  | Mean | 0.3274±0.0013 | 0.2004±0.0012 | 0.6486±0.0014 |
|  | PUGAN | 1^st^ fold | 0.2945 | 0.1969 | 0.7181 |
|  |  | 2^nd^ fold | 0.297 | 0.1993 | 0.7142 |
|  |  | 3^rd^ fold | 0.2962 | 0.1987 | 0.716 |
|  |  | 4^th^ fold | 0.2981 | 0.2001 | 0.7135 |
|  |  | 5^th^ fold | 0.295 | 0.1972 | 0.7172 |
|  |  | Mean | 0.2962±0.0015 | 0.1984±0.0014 | 0.7158±0.0019 |
|  | SegNet PU | 1^st^ fold | 0.4629 | 0.3578 | 0.4132 |
|  |  | 2^nd^ fold | 0.4653 | 0.3609 | 0.4101 |
|  |  | 3^rd^ fold | 0.4645 | 0.3597 | 0.412 |
|  |  | 4^th^ fold | 0.4662 | 0.3621 | 0.4093 |
|  |  | 5^th^ fold | 0.4638 | 0.3586 | 0.4127 |
|  |  | Mean | 0.4645±0.0013 | 0.3598±0.0017 | 0.4115±0.0017 |
|  | PUNet | 1^st^ fold | 0.9331 | 1.1982 | 0.3002 |
|  |  | 2^nd^ fold | 0.937 | 1.2035 | 0.2969 |
|  |  | 3^rd^ fold | 0.9352 | 1.2011 | 0.2987 |
|  |  | 4^th^ fold | 0.9381 | 1.2059 | 0.2958 |
|  |  | 5^th^ fold | 0.934 | 1.1993 | 0.2995 |
|  |  | Mean | 0.9355±0.0021 | 1.2016±0.0031 | 0.2982±0.0018 |
|  | MAPUNet | 1^st^ fold | 0.2769 | 0.1863 | 0.7408 |
|  |  | 2^nd^ fold | 0.2954 | 0.2058 | 0.7589 |
|  |  | 3^rd^ fold | 0.3012 | 0.2124 | 0.7643 |
|  |  | 4^th^ fold | 0.2837 | 0.1949 | 0.7465 |
|  |  | 5^th^ fold | 0.2833 | 0.1886 | 0.7505 |
|  |  | Mean | 0.2881±0.0099 | 0.1976±0.0112 | 0.7522±0.0094 |
