# Supplementary material for: MAPUNet: Multi-scale attention for InSAR phase unwrapping in mining areas
Source: PLoS One. 2026 May 26;21(5):e0331189. doi: 10.1371/journal.pone.0331189 (PMC13210142; doi:10.1371/journal.pone.0331189)
Supplement: S2 Appendix — (DOCX) [file pone.0331189.s002.docx]

# **S2 Appendix-The deep learning five-fold cross-validation results of large phase gradients.**

**S2 Table 2. The deep learning five-fold cross-validation results of large phase gradients.**

| Noise | Method | K-fold | M | MSE | SSIM |
| --- | --- | --- | --- | --- | --- |
| SNR=8 | ResUNet | 1^st^ fold | 0.4549 | 0.8038 | 0.8049 |
|  |  | 2^nd^ fold | 0.4637 | 0.8254 | 0.8123 |
|  |  | 3^rd^ fold | 0.4751 | 0.8672 | 0.821 |
|  |  | 4^th^ fold | 0.4682 | 0.8426 | 0.8146 |
|  |  | 5^th^ fold | 0.4778 | 0.8315 | 0.8189 |
|  |  | Mean±std | 0.4679±0.0092 | 0.8341±0.0233 | 0.8143±0.0063 |
|  | UNet++ | 1^st^ fold | 0.1961 | 0.1036 | 0.8729 |
|  |  | 2^nd^ fold | 0.1993 | 0.1061 | 0.8758 |
|  |  | 3^rd^ fold | 0.2022 | 0.1104 | 0.8799 |
|  |  | 4^th^ fold | 0.2011 | 0.1078 | 0.8764 |
|  |  | 5^th^ fold | 0.1997 | 0.1059 | 0.8786 |
|  |  | Mean | 0.1997±0.0023 | 0.1068±0.0025 | 0.8767±0.0027 |
|  | PUGAN | 1^st^ fold | 0.2104 | 0.1015 | 0.8789 |
|  |  | 2^nd^ fold | 0.2148 | 0.1049 | 0.8835 |
|  |  | 3^rd^ fold | 0.2189 | 0.1092 | 0.8879 |
|  |  | 4^th^ fold | 0.2141 | 0.1056 | 0.8846 |
|  |  | 5^th^ fold | 0.2157 | 0.1041 | 0.8838 |
|  |  | Mean | 0.2148±0.0031 | 0.1051±0.0028 | 0.8837±0.0032 |
|  | SegNet PU | 1^st^ fold | 0.2149 | 0.1272 | 0.7979 |
|  |  | 2^nd^ fold | 0.2194 | 0.1313 | 0.8019 |
|  |  | 3^rd^ fold | 0.2253 | 0.1372 | 0.8096 |
|  |  | 4^th^ fold | 0.2216 | 0.1336 | 0.8058 |
|  |  | 5^th^ fold | 0.2231 | 0.1355 | 0.8071 |
|  |  | Mean | 0.2209±0.004 | 0.133±0.0039 | 0.8045±0.0046 |
|  | PUNet | 1^st^ fold | 0.7712 | 1.0003 | 0.5946 |
|  |  | 2^nd^ fold | 0.7851 | 1.0186 | 0.6021 |
|  |  | 3^rd^ fold | 0.8027 | 1.0508 | 0.6124 |
|  |  | 4^th^ fold | 0.7906 | 1.0341 | 0.6063 |
|  |  | 5^th^ fold | 0.7862 | 1.0127 | 0.6004 |
|  |  | Mean | 0.7872±0.0113 | 1.0233±0.0196 | 0.6032±0.0067 |
|  | MAPUNet | 1^st^ fold | 0.2099 | 0.0946 | 0.8861 |
|  |  | 2^nd^ fold | 0.2114 | 0.0959 | 0.8872 |
|  |  | 3^rd^ fold | 0.2126 | 0.0968 | 0.8891 |
|  |  | 4^th^ fold | 0.2111 | 0.0962 | 0.8876 |
|  |  | 5^th^ fold | 0.2108 | 0.0957 | 0.8884 |
|  |  | Mean | 0.2112±0.001 | 0.0958±0.0008 | 0.8877±0.0011 |
| SNR=4 | ResUNet | 1^st^ fold | 0.4731 | 0.9245 | 0.7689 |
|  |  | 2^nd^ fold | 0.4802 | 0.9496 | 0.7724 |
|  |  | 3^rd^ fold | 0.4918 | 0.9836 | 0.7801 |
|  |  | 4^th^ fold | 0.4861 | 0.9721 | 0.7775 |
|  |  | 5^th^ fold | 0.4934 | 0.9702 | 0.7763 |
|  |  | Mean | 0.4849±0.0084 | 0.96±0.0233 | 0.775±0.0044 |
|  | UNet++ | 1^st^ fold | 0.2362 | 0.1291 | 0.8128 |
|  |  | 2^nd^ fold | 0.2381 | 0.131 | 0.8153 |
|  |  | 3^rd^ fold | 0.2412 | 0.1357 | 0.8215 |
|  |  | 4^th^ fold | 0.2391 | 0.1342 | 0.819 |
|  |  | 5^th^ fold | 0.2403 | 0.1324 | 0.8181 |
|  |  | Mean | 0.239±0.0019 | 0.1325±0.0026 | 0.8173±0.0034 |
|  | PUGAN | 1^st^ fold | 0.2218 | 0.1127 | 0.8589 |
|  |  | 2^nd^ fold | 0.2241 | 0.1143 | 0.8605 |
|  |  | 3^rd^ fold | 0.2286 | 0.1198 | 0.8653 |
|  |  | 4^th^ fold | 0.2267 | 0.1172 | 0.8637 |
|  |  | 5^th^ fold | 0.2261 | 0.1138 | 0.863 |
|  |  | Mean | 0.2255±0.0026 | 0.1156±0.0029 | 0.8623±0.0026 |
|  | SegNet PU | 1^st^ fold | 0.2741 | 0.1753 | 0.7005 |
|  |  | 2^nd^ fold | 0.2776 | 0.1791 | 0.7021 |
|  |  | 3^rd^ fold | 0.2879 | 0.1869 | 0.709 |
|  |  | 4^th^ fold | 0.2835 | 0.1816 | 0.7058 |
|  |  | 5^th^ fold | 0.2801 | 0.1787 | 0.7036 |
|  |  | Mean | 0.2806±0.0053 | 0.1803±0.0043 | 0.7042±0.0033 |
|  | PUNet | 1^st^ fold | 0.8368 | 1.0881 | 0.5529 |
|  |  | 2^nd^ fold | 0.8482 | 1.1017 | 0.5571 |
|  |  | 3^rd^ fold | 0.8764 | 1.1605 | 0.5681 |
|  |  | 4^th^ fold | 0.8576 | 1.1292 | 0.563 |
|  |  | 5^th^ fold | 0.8473 | 1.1189 | 0.5607 |
|  |  | Mean | 0.8533±0.0149 | 1.1197±0.0277 | 0.5604±0.0058 |
|  | MAPUNet | 1^st^ fold | 0.2166 | 0.0927 | 0.863 |
|  |  | 2^nd^ fold | 0.2173 | 0.0932 | 0.8635 |
|  |  | 3^rd^ fold | 0.218 | 0.094 | 0.8645 |
|  |  | 4^th^ fold | 0.2175 | 0.0936 | 0.864 |
|  |  | 5^th^ fold | 0.2164 | 0.0929 | 0.8633 |
|  |  | Mean | 0.2172±0.0007 | 0.0933±0.0005 | 0.8637±0.0006 |
| SNR=1 | ResUNet | 1^st^ fold | 0.6935 | 1.9568 | 0.6038 |
|  |  | 2^nd^ fold | 0.7047 | 1.9861 | 0.6061 |
|  |  | 3^rd^ fold | 0.7344 | 2.0812 | 0.6132 |
|  |  | 4^th^ fold | 0.7191 | 2.0346 | 0.6106 |
|  |  | 5^th^ fold | 0.6983 | 1.9725 | 0.6081 |
|  |  | Mean | 0.71±0.0167 | 2.0062±0.051 | 0.6084±0.0037 |
|  | UNet++ | 1^st^ fold | 0.4398 | 0.3462 | 0.5836 |
|  |  | 2^nd^ fold | 0.4436 | 0.3498 | 0.586 |
|  |  | 3^rd^ fold | 0.4499 | 0.3607 | 0.5921 |
|  |  | 4^th^ fold | 0.4455 | 0.3554 | 0.5889 |
|  |  | 5^th^ fold | 0.4441 | 0.3526 | 0.5895 |
|  |  | Mean | 0.4446±0.0036 | 0.3529±0.0055 | 0.588±0.0033 |
|  | PUGAN | 1^st^ fold | 0.3319 | 0.3299 | 0.7417 |
|  |  | 2^nd^ fold | 0.3342 | 0.3321 | 0.7434 |
|  |  | 3^rd^ fold | 0.3418 | 0.3409 | 0.7501 |
|  |  | 4^th^ fold | 0.3376 | 0.3358 | 0.7469 |
|  |  | 5^th^ fold | 0.3364 | 0.3314 | 0.7433 |
|  |  | Mean | 0.3364±0.0037 | 0.334±0.0044 | 0.7451±0.0034 |
|  | SegNet PU | 1^st^ fold | 0.4886 | 0.4201 | 0.4638 |
|  |  | 2^nd^ fold | 0.4919 | 0.4249 | 0.4666 |
|  |  | 3^rd^ fold | 0.5068 | 0.4413 | 0.4751 |
|  |  | 4^th^ fold | 0.4991 | 0.4342 | 0.4716 |
|  |  | 5^th^ fold | 0.4952 | 0.4273 | 0.4711 |
|  |  | Mean | 0.4963±0.007 | 0.4296±0.0083 | 0.4696±0.0044 |
|  | PUNet | 1^st^ fold | 1.0938 | 1.7369 | 0.4585 |
|  |  | 2^nd^ fold | 1.1175 | 1.7658 | 0.4607 |
|  |  | 3^rd^ fold | 1.1673 | 1.8496 | 0.4711 |
|  |  | 4^th^ fold | 1.1351 | 1.8063 | 0.4678 |
|  |  | 5^th^ fold | 1.1336 | 1.7927 | 0.465 |
|  |  | Mean | 1.1295±0.0269 | 1.7903±0.0425 | 0.4646±0.0051 |
|  | MAPUNet | 1^st^ fold | 0.2859 | 0.1653 | 0.7621 |
|  |  | 2^nd^ fold | 0.2864 | 0.1658 | 0.7625 |
|  |  | 3^rd^ fold | 0.2875 | 0.167 | 0.7638 |
|  |  | 4^th^ fold | 0.2869 | 0.1664 | 0.7631 |
|  |  | 5^th^ fold | 0.2861 | 0.1657 | 0.7622 |
|  |  | Mean | 0.2866±0.0006 | 0.166±0.0007 | 0.7627±0.0007 |
