# Supplementary material for: MAPUNet: Multi-scale attention for InSAR phase unwrapping in mining areas
Source: PLoS One. 2026 May 26;21(5):e0331189. doi: 10.1371/journal.pone.0331189 (PMC13210142; doi:10.1371/journal.pone.0331189)
Supplement: S3 Appendix — (DOCX) [file pone.0331189.s003.docx]

# **S3 Appendix-The specific means and standard deviations of small phase gradients.**

Here, the specific means and standard deviations are provided.

**S3 Table 3. The specific means and standard deviations of small phase gradients.**

| Noise | Method | M | MSE | SSIM |
| --- | --- | --- | --- | --- |
| SNR=8 | ResUNet | 0.3416±0.0019 | 0.3506±0.0018 | 0.8138±0.0018 |
|  | UNet++ | 0.1915±0.0012 | 0.1092±0.0007 | 0.8873±0.0014 |
|  | PUGAN | 0.1402±0.0013 | 0.0446±0.0008 | 0.9008±0.0015 |
|  | SegNet PU | 0.1711±0.001 | 0.0603±0.0006 | 0.7741±0.0013 |
|  | PUNet | 0.3846±0.0014 | 0.2473±0.0015 | 0.6892±0.0011 |
|  | MAPUNet | 0.1538±0.001 | 0.0476±0.0007 | 0.8915±0.0012 |
| SNR=4 | ResUNet | 0.3626±0.0012 | 0.3864±0.0018 | 0.7579±0.0012 |
|  | UNet++ | 0.1893±0.0009 | 0.0905±0.0006 | 0.8563±0.0008 |
|  | PUGAN | 0.1545±0.0012 | 0.0491±0.0007 | 0.8651±0.0011 |
|  | SegNet PU | 0.2373±0.0014 | 0.1024±0.001 | 0.672±0.001 |
|  | PUNet | 0.4493±0.0014 | 0.3389±0.0017 | 0.6404±0.0013 |
|  | MAPUNet | 0.1666±0.0011 | 0.0533±0.0007 | 0.8634±0.0012 |
| SNR=1 | ResUNet | 0.4863±0.0013 | 0.5302±0.002 | 0.543±0.0019 |
|  | UNet++ | 0.3274±0.0013 | 0.2004±0.0012 | 0.6486±0.0014 |
|  | PUGAN | 0.2962±0.0015 | 0.1984±0.0014 | 0.7158±0.0019 |
|  | SegNet PU | 0.4645±0.0013 | 0.3598±0.0017 | 0.4115±0.0017 |
|  | PUNet | 0.9355±0.0021 | 1.2016±0.0031 | 0.2982±0.0018 |
|  | MAPUNet | 0.2881±0.0099 | 0.1976±0.0112 | 0.7522±0.0094 |
