# Supplementary material for: MAPUNet: Multi-scale attention for InSAR phase unwrapping in mining areas
Source: PLoS One. 2026 May 26;21(5):e0331189. doi: 10.1371/journal.pone.0331189 (PMC13210142; doi:10.1371/journal.pone.0331189)
Supplement: S4 Appendix — (DOCX) [file pone.0331189.s004.docx]

# **S4 Appendix-The specific means and standard deviations of large phase gradients.**

Here, the specific means and standard deviations are provided.

**S4 Table 4. The specific means and standard deviations of large phase gradients.**

| Noise | Method | M | MSE | SSIM |
| --- | --- | --- | --- | --- |
| SNR=8 | ResUNet | 0.4679±0.0092 | 0.8341±0.0233 | 0.8143±0.0063 |
|  | UNet++ | 0.1997±0.0023 | 0.1068±0.0025 | 0.8767±0.0027 |
|  | PUGAN | 0.2148±0.0031 | 0.1051±0.0028 | 0.8837±0.0032 |
|  | SegNet PU | 0.2209±0.004 | 0.133±0.0039 | 0.8045±0.0046 |
|  | PUNet | 0.7872±0.0113 | 1.0233±0.0196 | 0.6032±0.0067 |
|  | MAPUNet | 0.2112±0.001 | 0.0958±0.0008 | 0.8877±0.0011 |
| SNR=4 | ResUNet | 0.4849±0.0084 | 0.96±0.0233 | 0.775±0.0044 |
|  | UNet++ | 0.239±0.0019 | 0.1325±0.0026 | 0.8173±0.0034 |
|  | PUGAN | 0.2255±0.0026 | 0.1156±0.0029 | 0.8623±0.0026 |
|  | SegNet PU | 0.2806±0.0053 | 0.1803±0.0043 | 0.7042±0.0033 |
|  | PUNet | 0.8533±0.0149 | 1.1197±0.0277 | 0.5604±0.0058 |
|  | MAPUNet | 0.2172±0.0007 | 0.0933±0.0005 | 0.8637±0.0006 |
| SNR=1 | ResUNet | 0.71±0.0167 | 2.0062±0.051 | 0.6084±0.0037 |
|  | UNet++ | 0.4446±0.0036 | 0.3529±0.0055 | 0.588±0.0033 |
|  | PUGAN | 0.3364±0.0037 | 0.334±0.0044 | 0.7451±0.0034 |
|  | SegNet PU | 0.4963±0.007 | 0.4296±0.0083 | 0.4696±0.0044 |
|  | PUNet | 1.1295±0.0269 | 1.7903±0.0425 | 0.4646±0.0051 |
|  | MAPUNet | 0.2866±0.0006 | 0.166±0.0007 | 0.7627±0.0007 |
