# Supplementary material for: MAPUNet: Multi-scale attention for InSAR phase unwrapping in mining areas
Source: PLoS One. 2026 May 26;21(5):e0331189. doi: 10.1371/journal.pone.0331189 (PMC13210142; doi:10.1371/journal.pone.0331189)
Supplement: S5 Appendix — (DOCX) [file pone.0331189.s005.docx]

# **S5 Appendix-Generalization experiment (Location of the study area)**

**Generalization experiment**

**Location of the study area**

The Hami mining area (92°30′ - 93°50′ E, 42°10′ - 42°30′ N) is located within the Nanhui area of Yizhou District, Hami City, Xinjiang Province. The Hami region has a temperate continental climate and is arid. It is higher in the north and lower in the south, with the southern part being relatively flat. This area is rich in mineral resources, with 76 types of minerals having been identified, and some of these have reserves ranking first in the entire Xinjiang. The study area is shown in S2 Fig. 1 (a), and the red box represents the main research area of this paper, which is mainly located in Yizhou District of Hami. S2 Fig. 1 (b) is the optical image covering this area.

| 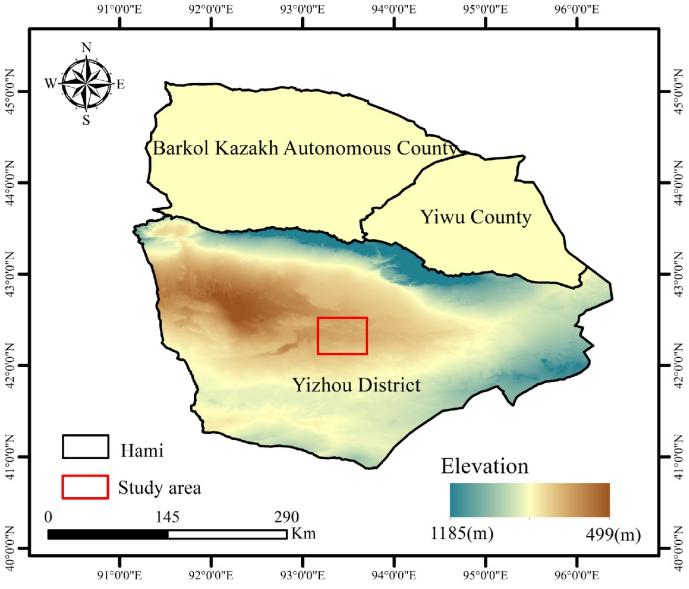 | 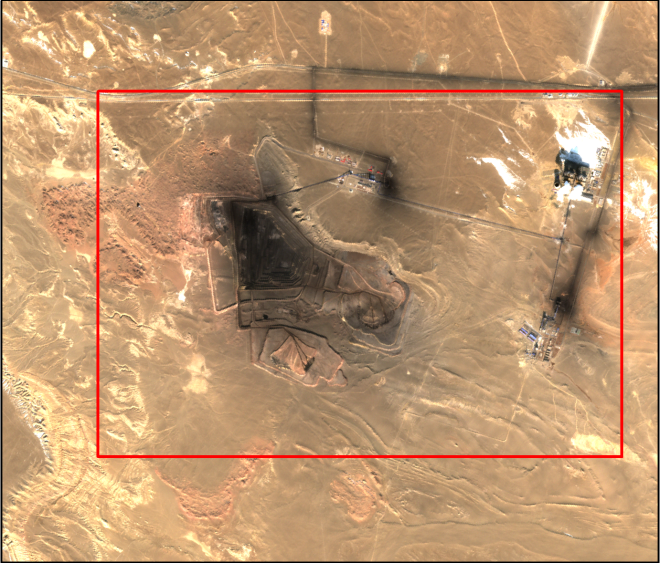 |
| --- | --- |
| （a）Hami Research Area | （b）Optical image map of the study area |

**S5 Fig. 1 Location of the Hami study area.**
