# Supplementary material for: MAPUNet: Multi-scale attention for InSAR phase unwrapping in mining areas
Source: PLoS One. 2026 May 26;21(5):e0331189. doi: 10.1371/journal.pone.0331189 (PMC13210142; doi:10.1371/journal.pone.0331189)
Supplement: S6 Appendix — (DOCX) [file pone.0331189.s006.docx]

# **S6 Appendix-Generalization experiment experiment (Experimental Data)**

**Generalization experiment**

**Experimental Data**

Four ascending orbit Sentinel-1A images from January 2020 to April 2022 were selected and composed into three interferometric pairs. The specific information of the interferometric pairs is shown in S2 Table 1.

**S6 Table 5. Information of Sentinel-1A interferometric pairs.**

| No. | Master image | Slave image | Relative orbit | Frame | Temporal baseline(d) |
| --- | --- | --- | --- | --- | --- |
| Hami | 2020/01/10 | 2020/05/21 | 48 | 449 | 132 |
|  | 2020/01/10 | 2021/05/16 | 48 | 449 | 492 |
|  | 2020/01/10 | 2022/04/17 | 48 | 449 | 828 |
