# Supplementary material for: MAPUNet: Multi-scale attention for InSAR phase unwrapping in mining areas
Source: PLoS One. 2026 May 26;21(5):e0331189. doi: 10.1371/journal.pone.0331189 (PMC13210142; doi:10.1371/journal.pone.0331189)
Supplement: S7 Appendix — (DOCX) [file pone.0331189.s007.docx]

# **S7 Appendix-Generalization experiment (Phase Unwrapping Results and Accuracy Validation)**

**Generalization experiment**

**Phase Unwrapping Results and Accuracy Validation**

The MAPUNet model was selected for PU. S2 Fig. 2 shows the unwrapped and rewrapped phase results for the mining area across three time periods. As can be seen from (a) to (c), the proposed method successfully unwraps the phase for the Hami mining area. The unwrapped results were rewrapped, and the interferograms of the subsidence basins in the resulting wrapped image were clear and continuous. This proves that the method proposed in this paper can effectively solve the problem of severe confusion of interferometric fringes in subsidence basins of the long temporal baselines in the Hami mining area, and achieves high-quality unwrapping results.

| 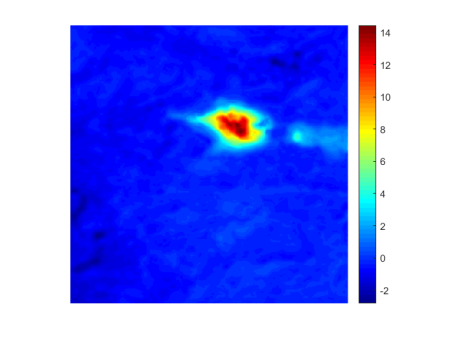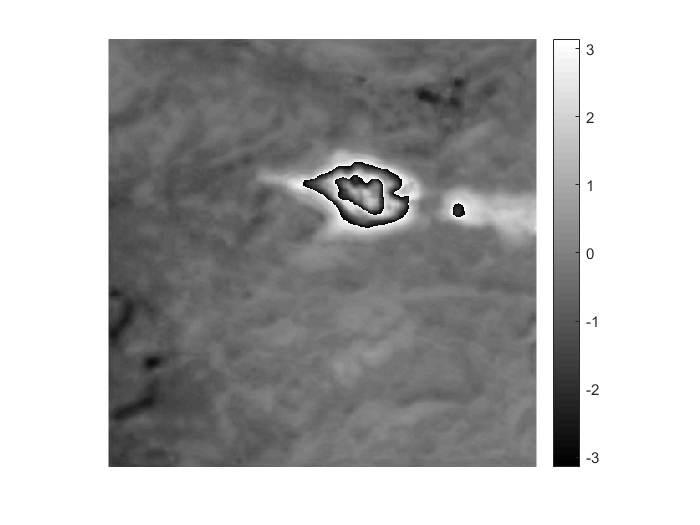  (a) From 2020-01-10 to 2020-05-21 | 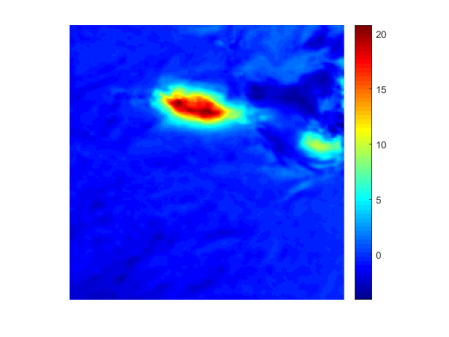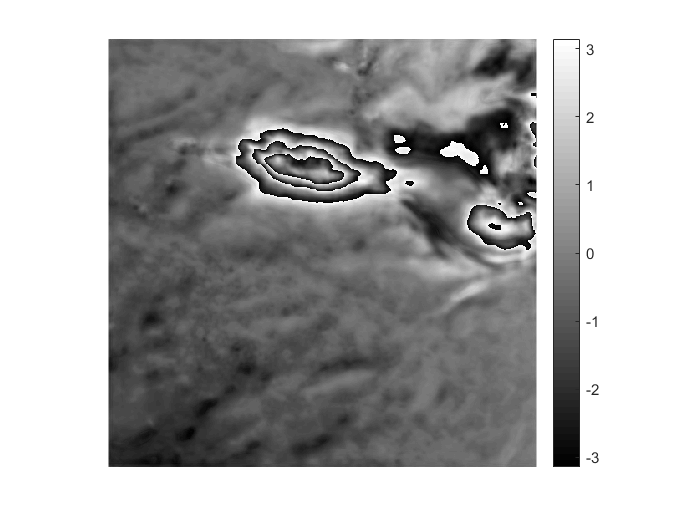  (b) From 2020-01-10 to 2021-05-16 |
| --- | --- |
| 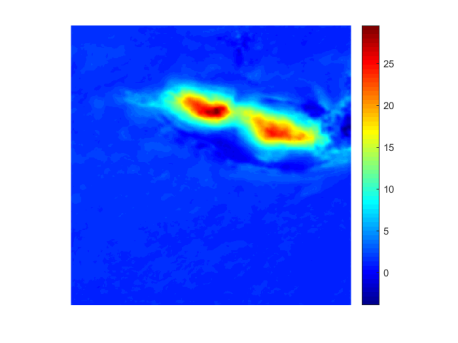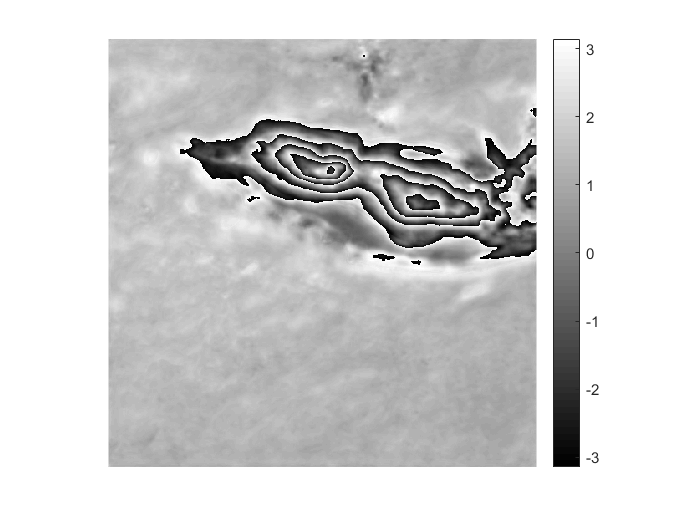  (c) From 2020-01-10 to 2022-04-17 | |

**S7 Fig. 2 The unwrapping and rewrapped results of Hami mining.**
